# A case of secretory carcinoma of the submandibular gland with unusual immunohistochemical staining

**DOI:** 10.1002/ccr3.2927

**Published:** 2020-06-08

**Authors:** Alexander Karabachev, Ricardo Aulet, Mirabelle Sajisevi, Allison Ciolino

**Affiliations:** ^1^ Robert Larner College of Medicine University of Vermont Burlington VT USA; ^2^ Department of Surgery Division of Otolaryngology University of Vermont Medical Center Burlington VT USA; ^3^ Department of Pathology and Laboratory Medicine University of Vermont Medical Center Burlington VT USA

**Keywords:** acinar cell carcinoma, carcinoma, head and neck neoplasms, salivary gland, submandibular gland

## Abstract

Mammaglobin negative secretory carcinoma may be overlooked. It is important to assess the possibility of diagnosis when histology is suggestive and immunohistochemical staining for S‐100 is positive even when staining for mammaglobin is negative.

## Graphical abstract

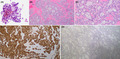

## 1 INTRODUCTION

In 2010, Skálová et al first described a series of salivary gland carcinomas with histologic resemblance to secretory breast cancer referred to as mammary analogue secretory carcinoma (MASC) and most recently referred to as secretory carcinoma (SC) of the salivary gland according to the WHO Classification of Head and Neck Tumours. Both secretory breast cancer and SC of the salivary gland harbor the balanced chromosomal translocation *t*(12;15)(p13:q25) with the resulting *ETV6‐NTRK3* fusion.^1^ Prior to this discovery, SC was often incorrectly diagnosed as zymogen‐poor acinic cell carcinoma and adenocarcinoma not otherwise specified; therefore, the recognition of SC led to the reexamination of several groups of salivary gland tumors.^2^, ^3^, ^4^ A subsequent study noted that 79% of alleged acinic cell carcinomas of nonparotid origin represent misclassified SCs.^5^ SC may be distinguished from other salivary gland tumors on the basis of an *ETV6* translocation together with strong staining for mammaglobin and S‐100. Pinto et al showed mammaglobin positivity to be 100% sensitive for SC of the salivary gland^6^; however, it may also be positive in other salivary gland tumors.^7^ Here, we describe a case of SC of the submandibular gland with negative mammaglobin staining.

## 2 CASE REPORT

**FIGURE 1:**
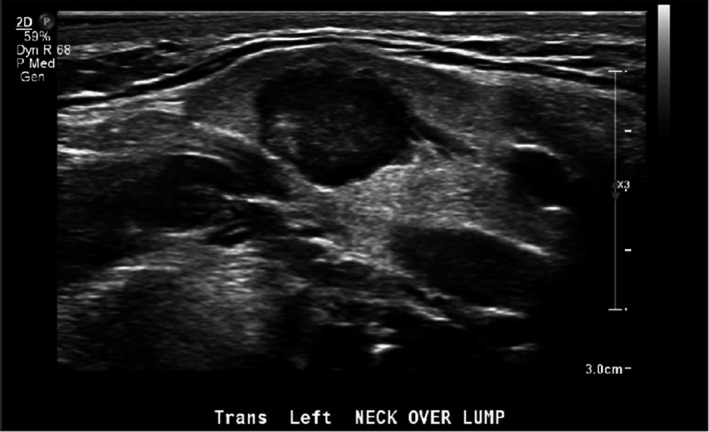
Ultrasound of the left submandibular gland showing a 1.6 × 1.2 × 1.3 centimeter irregularly marginated, hypovascular, and hypoechoic lesion

An 18‐year‐old male with no prior medical history was referred by his primary care physician to otolaryngology for evaluation of a lump in the left side of his neck that had been present for greater than a year with slight enlargement. He described it as firm and occasionally painful; however, it had been mostly asymptomatic with no lesions on the skin or drainage. Patient denied having fevers, chills, night sweats, fatigue, and weight loss. Ultrasound showed a 1.6 × 1.2 × 1.3 centimeter irregularly marginated, hypovascular, and hypoechoic lesion in the left submandibular gland (Figure 1). Adjacent normal‐sized lymph nodes were observed, and evaluation of the contralateral submandibular gland revealed a normal gland with no lesions.

**FIGURE 2:**
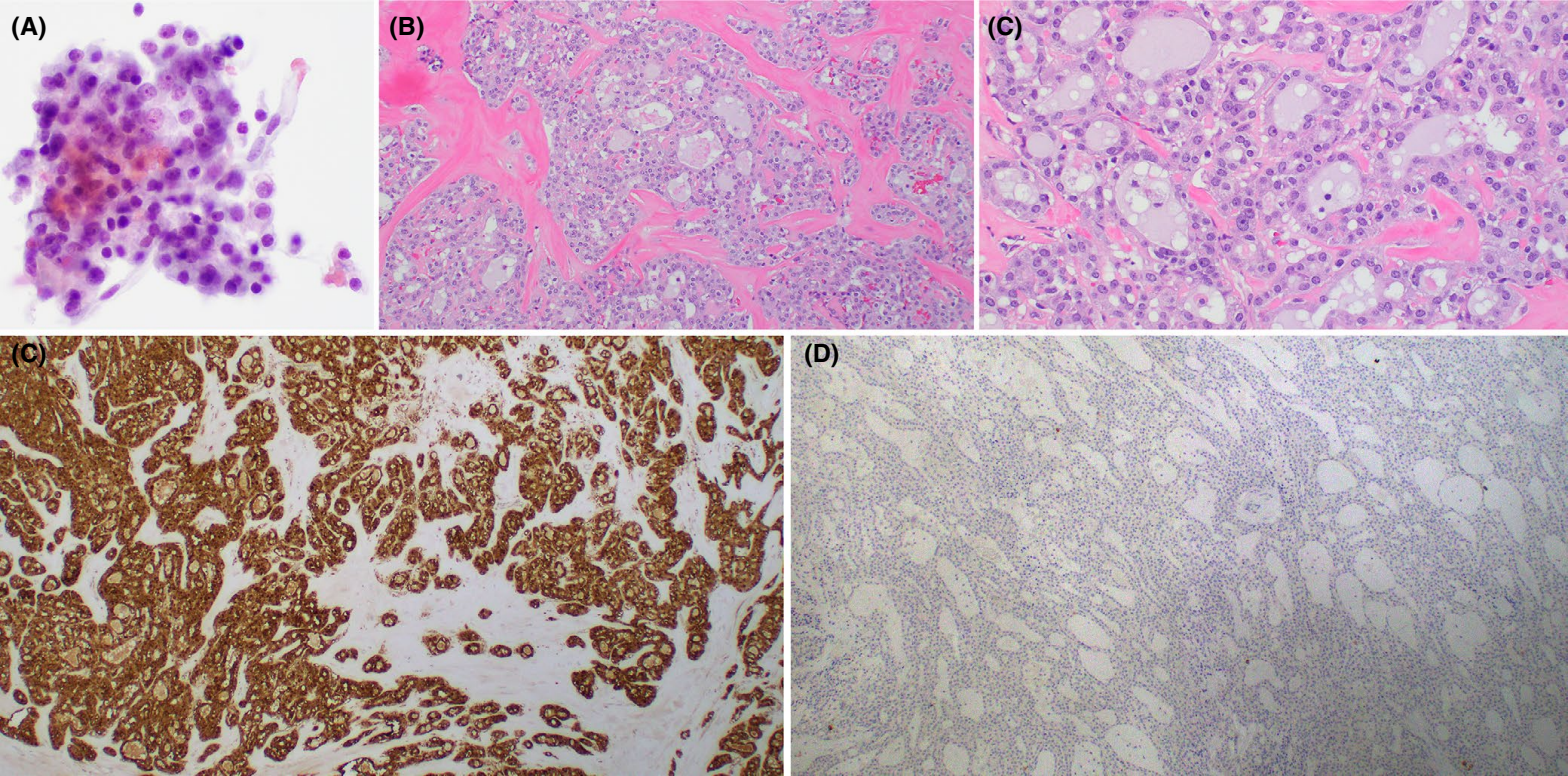
A, Fine‐needle aspiration of left submandibular gland shows a monotonous population of atypical cells with round nuclei, distinct nucleoli, and moderate amounts of granular to vacuolated cytoplasm (×400). B, Histologic sections demonstrate a low‐grade salivary gland malignancy arranged in lobules separated by a dense sclerotic stroma with tubular and microcystic growth formation (×100). C, Higher magnification highlights uniform round nuclei, small distinct nucleoli, and vacuolated cytoplasm lacking intracytoplasmic zymogen granules. There is no significant nuclear pleomorphism, mitosis, or necrosis identified (×200). D, Tumor cells show strong diffuse immunoreactivity for S‐100 (40×). E, Tumor cells show complete absence of staining for mammaglobin (40×)

Fine‐needle aspiration (FNA) revealed a hypocellular monotonous population of atypical cells with distinct nucleoli and vacuolated cytoplasm, arranged in crowded groups (Figure 2A). Based on these findings, a salivary gland neoplasm could not be excluded and it was decided to proceed with excision of the gland for definitive diagnosis and treatment. Patient underwent a left submandibular gland excision, and the specimen was sent for routine pathology. Final pathology revealed a low‐grade adenocarcinoma with microcystic and tubular growth comprised of cells with round nuclei, fine chromatin, distinct nucleoli, and eosinophilic granular to vacuolated cytoplasm (Figure 2B,C). The tumor infiltrated into submandibular parenchyma and demonstrated perineural invasion although surgical margins were negative. Immunohistochemical (IHC) staining was performed and showed diffuse positivity for S‐100 (Clone 4C4.9, Ventana) (Figure 2D), CK7 (Clone RN7, Leica), GATA‐3 (Clone L50‐823, Ventana), and SOX‐10 (Clone EP268, Epitomics) with focal positivity for p63 (Clone 4A4, Ventana) in a nonbiphenotypic pattern. The tumor cells showed complete absence of staining for mammaglobin (Clone 31A5, Leica; Figure 2E) and DOG‐1 (Clone K9, Leica). The morphologic features in conjunction with diffuse GATA‐3 and S‐100 positivity raised the possibility of SC; however, the complete absence of mammaglobin positivity was unusual.

**FIGURE 3:**
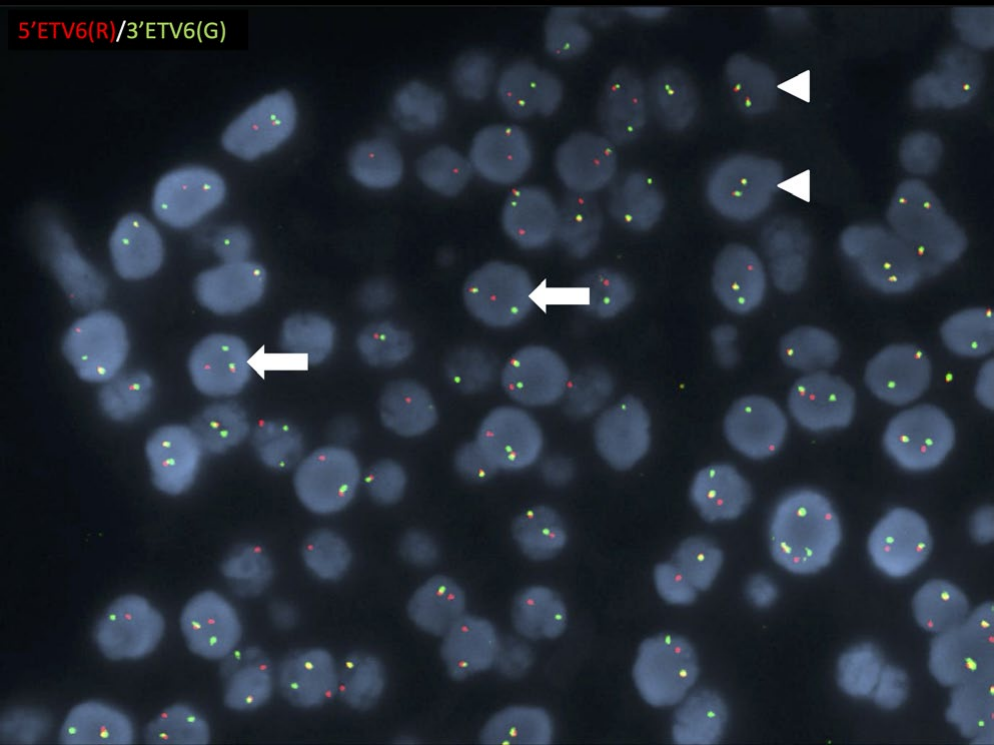
Fluorescence in situ hybridization (FISH) analysis using the ETV6 Dual Color Break Apart Probe demonstrates interphase nuclei with disrupted ETV6 signal, indicating rearrangement of the ETV6 gene at 12p13.2 locus utilizing 5'ETV6 (red) probe and 3'ETV6 (green) probe. In this case, 86 of 100 nuclei were scored as disrupted and are depicted in the above image by separation (break apart) of red and green signals (arrows). Rare cells with intact, nondisrupted signals (arrowheads) are also depicted

Given these unusual findings, the case was sent for consultative review to the Department of Anatomic Pathology at the Moffitt Cancer Center. Repeat immunohistochemical staining, performed at the Moffitt Cancer Center, for S100 and mammaglobin, showed similar findings. Subsequent fluorescence in situ hybridization (FISH) analysis, performed at Mayo Clinic Laboratories, was positive for the rearrangement of the *ETV6* gene at 12p13.2 (Figure 3) Histologic appearance of the lesion in conjunction with *ETV6* gene rearrangement was consistent with the diagnosis of SC of the salivary gland even in the context of mammaglobin negativity. Given the low‐grade morphology with complete excision, recommendations were for close observation with no additional treatment. Eleven months after surgery, the patient reports he is doing well with no specific concerns regarding his salivary gland tumor.

## 3 DISCUSSION

SC of the salivary gland is a newly listed entity in the 4th edition of the WHO classification of head and neck tumors with histologic resemblance to secretory breast cancer first described by Skalova et al in 2010. Many subsequent cases and studies have been published reassigning previously diagnosed salivary gland tumors to SC. Since its discovery, the histologic features, immunohistochemistry, clinical outcomes, and demographics have been thoroughly studied.^3^, ^8^, ^9^, ^10^

Although SC of the salivary gland consistently harbor rearrangements of the ETV6 gene at 12p13.2 resulting in ETV6 gene fusions, most commonly involving the NTRK3 gene *t*(12;15)(p13;q25), experts have now recognized that the diagnosis of SC may be rendered in the presence of classic histomorphology with dual positivity for mammaglobin and S‐100 immunostains, independent of molecular testing.^5^ Histologically, SC of the salivary gland is architecturally variable and may demonstrate back to back tubules or microcysts, papillae, or macrocysts. The tumor cells contain granular eosinophilic cytoplasm which lacks zymogen granules and often have a vacuolated or “bubbly” appearance with some similarities to apocrine cells.^11^ IHC staining is a very useful technique for distinguishing SC from other salivary gland tumors. The high sensitivity for diffuse S‐100 positivity in SC is a useful diagnostic characteristic, particularly since S‐100 staining in acinic cell carcinoma has been reported to be positive in only 10% of cases.^12^, ^13^ Similar to secretory breast carcinoma, SC of the salivary gland is positive for mammaglobin and has demonstrated a very high sensitivity. According to an analysis by Pinto et al in 2014 of five previously published case series and case reports, mammaglobin was positive in all 39 cases of SC of the salivary gland demonstrating 100% sensitivity. Only one study published from University of Campinas in Campinas, Brazil, in 2013 described mammaglobin‐negative SC through the analysis of 10 salivary gland tumors with *ETV6* translocation and found that 3/10 stained negative for mammaglobin.^14^ In addition, diffuse GATA‐3 expression has shown to provide further immunohistochemical support for the diagnosis of SC and can be utilized in the panel of stains for immunohistochemical investigation.^15^ Although focal positivity for GATA‐3 can be seen in several salivary gland tumors, diffuse positivity as defined by Schwartz et al as ≥50% in salivary gland tumors (PMID: 23604756) is limited to secretory carcinoma and salivary duct carcinoma.

The development of specific treatment options for SC compared to other salivary gland tumors enhances the importance of correct and timely diagnosis. Currently, there is an ongoing Phase 1 study, STARTRK‐2 (Studies of Tumor Alterations Responsive to Targeting Receptor Kinases)‐2, evaluating entrectinib, a tyrosine kinase inhibitor, and its treatment response in MASC with an estimated primary completion date of December 2022.^16^ Also, Drillon et al in 2018 demonstrated larotrectinib, a highly selective TRK inhibitor, to have antitumor activity in patients with TRK fusion‐positive cancer (ClinicalTrials.gov numbers, NCT02122913, NCT02637687, and NCT02576431).^17^

In this case, the histomorphology and the IHC staining for S‐100 and GATA‐3 would suggest a diagnosis of SC; however, due to the complete absence of mammaglobin positivity, the diagnosis could not be confirmed without testing for rearrangement of the *ETV6* gene at the 12p13.2 locus. This case was further complicated by positive SOX‐10 and focal p63 immunostaining, neither of which are classic findings for MASC.^7^ In summary, when histomorphology and IHC staining for S‐100 are suggestive for SC, however, mammaglobin staining is negative, molecular methods should be used to confirm the diagnosis to ensure proper management of this tumor.

## 4 CONCLUSION

SC of the salivary gland with negative mammaglobin staining can be overlooked. Targeted gene therapy has shown potential for the treatment of SC signifying added importance for ensuring the proper diagnosis. Otolaryngologists should be aware of this pitfall and work with their pathologists to further assess the possibility of SC when the histology is suggestive and S‐100 is positive even in the context of negative mammaglobin staining.

### 4.1 Meeting Information

Triological Society Combined Sections Meeting**,** Coronado, California, USA, January 23‐25, 2020.

## AUTHOR CONTRIBUTIONS

AK: performed the literature review, and wrote and submitted the manuscript. RA: involved in the treatment of the patient and provided oversight and edits to the writing and submission of the manuscript and the literature review. MS: involved in the treatment of the patient and provided oversight and edits to the writing and submission of the manuscript and the literature review. AC: involved in the treatment of the patient and provided oversight and edits to the writing and submission of the manuscript, the literature review, and preparing the pathology images for submission.

## CONFLICTS OF INTEREST

None.
